# Editorial

**Published:** 1864-01

**Authors:** 


### Editorial.

### A CASE.

Mr. O—, aged 30 years, of good health, of sanguine nervous temperament; on the 5th of January requested an examination of the first right inferior molar, which had given him intense pain for several days. It began in the tooth, and extended along the jaw, and somewhat over the side of the face and head, partaking somewhat of a neuralgic character.

The tooth had on its anterior proximal surface, a filling of medium size, which had been inserted about three years before; the pulp was then not exposed, nor was the dentine much sensitive; no unfavorable symptoms had been experienced, till the occurrence of this difficulty.

The pain was paroxysmal, and of great intensity. Entire quie was enjoyed during the intervals; there was no soreness of the tooth under percussion, showing that the difficulty was not in the periosteum, but that it was with the pulp and its ramifications, at least this was the conclusion.

Though the case was rather obscure in its cause and condition, the conclusion was to make an opening into the pulp-chamber, not through the filling, for that was in good condition, but through the central depression of the grinding surface of the tooth, this being the shortest and most direct route into the chamber. The opening being made, the pulp was intensely sensitive. An application of cobalt was made, for the purpose of destroying the pulp, which was accomplished in about twenty-four hours.

There was no pain after the application was made. The cavity and canals were cleaned out and filled, and all is well since; and the presumption is, will continue so.

This case is not presented because it is a rare one, for such occur frequently. Such cases present great difficulties, especially to the inexperienced; and, indeed to many whose professional age ought to give them more experience than they really possess.

We give this case, however, to refer to the fact, that temporizing and paliative treatment in such cases are wholly inefficient; they usually protract the suffering of a patient, and oftentimes involve the loss of a valuable tooth. Sometimes, from such a cause only, what is denominated facial neuralgia will rage for a long time with great fury, and especially if not relieved by the formation of alveolar abscesses.

The question here arises, how or why do such cases occur The pulp was not exposed by decay,—it was wholly intact by the decay and the operation of filling, except so far as it could have been influenced through a considerable thickness of the dentine. We will only venture a few suggestions, rather than a direct reply to the question.

In all cases where a tooth is attacked by caries, the pulp receives an impression thereby, to a greater or less extent; that impression usually stimulates the pulp to a vigorous elaboration and deposition of bone material upon the walls of its chamber, thereby endeavoring to shield itself from exposure. This process is not always accomplished; but instead, the pulp is brought to the verge of disease; and thus it may stand, until some systemic change occurs that will induce actual disease in the tissue. This susceptibility to disease may exist in a great many cases, and be developed in but few. Everyone's path, perhaps, occasionally leads them just to the point of disease, and yet they are led away from it without feeling its touch.

The development of difficulties exhibited in cases of this kind, are dependent upon many things,—for instance, the general health or local affections, that may, by transfer or sympathy, exercise an influence or change in nourishment, etc.

Is there any treatment that will anticipate and prevent such results as these cases exhibit? Will saturating the dentine with creosote, previous to filling, avail anything? We think it will; how much, we are not prepared to say. Will burnishing the walls of the cavity result in any good? If so, upon what principle? Will any advantage be derived from escharotics?

We wish something further upon this subject. T.

### MEETINGS OF SOCIETIES.

American Dental Convention.—The tenth annual session will be held at Detroit, Mich., commencing Tuesday, August 2d, 1864.

*Order of Business.*—1. Reading the Constitution, and admission of members; 2. Reading the minutes of last Convention; 3. Reports of officers and standing committees; 4. Election of officers; 5. Retiring President's Address; 6. Induction of officers; 7. Reports of special committees; 8. Miscellaneous business.

*Order of Discussion.*—1. The best means of improving the practice and elevating the profession of dentistry; 2. Anæsthetics—their proper use and relative value; 3. Extracting teeth: when it should be done and when not,—the best instruments for the purpose and the subsequent treatment, when any is required 4. Absorption of alveolar process—causes and treatment; 5. Filling teeth: the relative value of different materials, and the mode of operating in difficult cases; 6. The best method of obtaining accurate impressions and models of the mouth; 7. The relative value of different materials as a base for artificial teeth; 8. Miscellaneous.

All written communications must be read to open the discussion of the subjects to which they relate, and must not occupy more than fifteen minutes in the reading.

No member shall speak more than ten minutes at one time, nor more than twice on the same subject, without the unanimous consent of the Convention.

The subjects selected for discussion are unusually practical, and are designed to elicit the results of actual experience and observation, rather than theories and speculations, which are better for the seclusion of the study than for public assemblies.

All dentists in regular practice may become members of the Convention, and all such are hereby invited to attend.

L. W. Rogers, Utica, N. Y.,

A. W. Kingsley, Elizabeth, N. J.,

J. A. Watling, Ypsilanti, Mich.,

A. Hill, Norwalk, Conn.,

H. A. Smith, Cincinnati, Ohio,

*Executive Committee.*

---

Mississippi Valley Dental Society.—The next annual meeting will take place in this city, at the lecture room of the Ohio College of Dental Surgery, on Wednesday, February 17th, at 10 o'clock A. M. At which time we hope to meet all the members of that Association. We anticipate a full meeting, and expect a good time. We say to all—come. T.

---

The Dental College Association—Will hold its regular annual meeting on Friday, February 19th, at 10 o'clock A. M., in the lecture room of the College. It is desired and expected, that most if not all of the stockholders be present, as there is important business to be transacted. Several matters intimately connected with the interest of the School must be considered and acted upon; hence it is very important that as many as possible be present. T.

